# The 5/95 gap in the indexation of psychiatric journals of low- and middle-income countries

**DOI:** 10.1111/j.1600-0447.2009.01476.x

**Published:** 2010-02

**Authors:** J J Mari, V Patel, C Kieling, D Razzouk, P Tyrer, H Herrman

**Affiliations:** 1 Department of Psychiatry, Universidade Federal de São PauloSão Paulo, SP, Brazil; 2 Health Services and Population Research Department, Institute of Psychiatry, King’s College, University of LondonLondon, UK; 3 Sangath CentrePorvorim, Goa, India; 4 London School of Hygiene and Tropical MedicineLondon, UK; 5 Department of Psychiatry, Hospital de Clinicas de Porto Alegre, Universidade Federal do Rio Grande do SulPorto Alegre, Brazil; 6 Department of Psychological Medicine, Imperial CollegeLondon, UK; 7 Orygen Youth Health Research Centre; 8 Secretary for Publications, World Psychiatric Association, The University of MelbourneMelbourne, Vic., Australia

**Keywords:** indexed psychiatric journals, low- and middle-income countries, psychiatric research, dissemination

## Abstract

Mari JJ, Patel V, Kieling C, Razzouk D, Tyrer P, Herrman H. The 5/95 gap in the indexation of psychiatric journals of low- and middle-income countries.

**Objective:**

To investigate the relationship between science production and the indexation level of low- and middle-income countries (LAMIC) journals in international databases.

**Method:**

Indicators of productivity in research were based on the number of articles produced over the 1994–2004 period. A survey in both Medline and ISI/Thomson was conducted to identify journals according to their country of origin. A WPA Task Force designed a collaborative process to assess distribution and quality of non-indexed LAMIC journals.

**Results:**

Twenty LAMIC were found to present more than 100 publications and a total of 222 indexed psychiatric journals were found, but only nine were from LAMIC. The Task Force received 26 questionnaires from editors of non-indexed journals, and concluded that five journals would meet criteria for indexation.

**Conclusion:**

Barriers to indexation of journals contribute to the difficulties in achieving fair representation in the main literature databases for the scientific production in these countries.

## Significant outcomes

1. At the time of the search, there were 222 mental health journals indexed in the Medline and/or ISI/Thomson, but only nine (4.1%) were from low- and middle-income countries (LAMIC).
2. Not a single low-income country journal was indexed.
3. None of the LAMIC in Asia and Africa has a journal indexed in the main international databases despite indications of a good level of scientific production in at least five of these countries, and even though several mental health journals in Africa and Asia apparently meet criteria for indexation.

## Limitations

1. The scientific production of LAMIC was based on the ISI count from the Psychiatry/Psychology section; criteria used by ISI/Thomson are not clearly defined, and there is a small chance of misclassification of subjects, and/or countries of origin.
2. It is possible that mental health articles published in non-psychiatric journals, such as journals of public health and general medicine, were missed in the counting. Also, it is likely that the list of non-indexed journals is incomplete; there may be many other LAMIC journals to be added to the list.
3. The replies to the questionnaires sent to editors may not be representative of the non-indexed journals, although the idea here was to identify the best available journals, a task most likely to have been achieved in our study.

## Introduction

Mental health publications from low- and middle-income countries (LAMIC) constitute a small proportion of the total research output in internationally accessible literature (1). Publications from LAMIC are poorly represented in mainstream psychiatric journals (2), and whenever available they are mostly led by authors from high-income countries (3), and fail to address public health needs (4). LAMIC are even more under-represented in high-impact psychiatric journals than they are in all international scientific journals (5). This systematic bias in the medical and psychiatric literature (6), where the health priorities of the developed world are taken for granted by editors of international journals, has been labeled institutional racism (7). Nonetheless, studies conducted in LAMIC are essential to cover the full spectrum of psychiatric disorders (6). In addition, locally driven research provides relevant information to guide policymakers in the expansion of cost effective and culturally adapted health services (8).

A major obstacle to disseminating LAMIC research is the scarcity of indexed journals with a strong LAMIC focus. The two most extensively recognized international databases of the medical and biomedical literature are the Medline/Pubmed and the Institute for Scientific Information ISI/Thomson Reuters (9).

### Aims of the study

A WPA task force was established in 2008 (including the authors JJM, VP, CK, PT and HH) to promote dissemination of mental health research in LAMIC. The task force began with studies aiming to: i) appraise the quality of the non-indexed LAMIC psychiatric journals to find potential candidates for indexation in the main databases; ii) assess the barriers to indexation of these journals; iii) identify the most productive LAMIC in Africa, Asia, Eastern Europe and Latin America and iv) investigate the relationship between research capacity and the indexation of LAMIC journals in the main databases.

## Material and methods

### The ISI essential indicators

The indicator of research productivity used to compare the development of mental health research among countries was the total number of papers for each LAMIC appearing in the Psychiatry/Psychology section of the ISI Essential Science Indicators database of Thomson Reuters from 1994 to 2004, ranked by countries. The study should include at least one author with an address from a LAMIC country, and the presence of two LAMIC authors in the same paper would be counted for both countries. All LAMIC were investigated and those presenting more than 100 publications in the period, an arbitrary operational criterion of scientific productivity, were selected for further scrutiny (from 1 January 1994 to 18 May 2004).

### Economic and health service indicators

Indicators – including gross domestic product (GDP), research and development expenditures and health expenditures – were extracted from the World Fact Book (10), the Human Development Indicators (11) and the Mental Health Atlas Project (12).

### The journals search

A survey in both Medline and ISI/Thomson databases was conducted to identify journals in the field of psychiatry according to their country of origin. The search in Medline was done by entering the expressions ‘psychiatry’ and ‘substance abuse’ in the journals database. All journals listed in Science and Social Sciences editions of the JCR for the category Psychiatry were included. Further details of the methodology can be seen elsewhere (13).

A preliminary list of non-indexed journals was derived from a report commissioned by WHO and the Global forum for Health Research (GFHR), on the basis of a hand search of the gray literature from those countries without publications either in Medline or PsycInfo (14). This list was then submitted to the 18 WPA Zonal Representatives, who were asked to identify, with the help of their Member Societies, journals published in the Zone countries, but missing from the list.

### Assessing the quality of non-indexed LAMIC psychiatric journals

Editors of non-indexed journals from LAMIC in Africa, Asia, Eastern Europe and Latin America, were invited to complete a specific questionnaire to provide information on the following points: i) the geographical representation of the journal; ii) the affiliation to a professional mental health society; iii) periodicity; iv) the composition of national and international editorial boards; v) the number of original and review articles in publications; vi) the language of publication for original articles and abstracts; vii) the level of current indexation; and viii) the availability of a friendly access website.

## Results

Twenty LAMIC countries (Table 1) were found to present more than 100 counted publications in the ISI Essential Science Indicators: one from Africa, seven from Asia, nine from Eastern Europe and three from Latin America. There are at least 78 non-indexed journals published in these countries, including three in South Africa, 16 in Asia, 11 in Central and Eastern Europe and 48 in Latin America. The smaller number of indexed journals includes five in Central and Eastern Europe and four in Latin America.

**1:** The distribution of publications for 20 selected low- and middle-income countries (LAMIC), the number of indexed and non-indexed journals, and the LAMIC social and economical characteristics

	Number of publications in ISI per million inhabitants (psychiatry) 1994–2004†	Number of indexed journals‡ (non-indexed journals)	GDP/per capita U$ dollars§	GERD/GDP¶	Health research expenditure as % total health expenditure††	Health expenditure as % of GDP‡‡	Number of psychiatrists per 100 000‡‡
Africa							
South Africa	21.6	0 (3)	13 000	0.7	1.0	8.6	1.2
Asia							
China	0.78	0 (14)	7600	1.2	1.0	5.5	1.3
India	0.43	0 (1)	3700	0.8	0.8	5.1	0.2
Taiwan	0.02	0 (1)	27 720	2.3	NA	6.2	NA
Republic of Korea	7.55	0 (0)	24 200	2.7	2.0	6.0	3.5
Singapore	64.7	0 (0)	49 900	2.3	NA	3.9	2.3
Kuwait	40.45	0 (0)	55 900	0.4	NA	3.9	3.1
United Arab Emirates	21.86	0 (0)	37 000	NA	NA	3.5	2.0
East-Central – Europe							
Russia	10.27	1 (0)	14 800	1.3	0.3	5.4	13.3
Turkey	7.13	1 (2)	12 000	0.64	1.0	5.0	1.0
Czech Republic	47.06	0 (1)	24 500	1.3	1.6	7.4	12.1
Greece	44.68	0 (0)	30 600	0.64	0.7	4.5	2.1
Slovakia	80.66	0 (0)	20 200	0.65	0.8	5.7	10
Hungary	25.07	1 (3)	19 300	0.95	2.0	6.8	9.0
Poland	6.75	1 (1)	16 200	0.67	1.6	6.1	6.0
Croatia	31.17	1 (4)	15 500	1.0	NA	9.0	8.7
Estonia	82.63	0 (0)	21 800	NA	NA	4.5	2.1
Latin America							
Mexico	7.83	1 (4)	10 600	0.4	1.3	6.1	2.8
Brazil	2.57	2 (18)	8600	1.0	1.5	7.6	4.8
Argentina	3.90	1 (1)	15 000	0.4	0.7	9.5	13.6

ISI, Institute for Scientific Information; GDP, gross domestic product; GERD, gross domestic expenditure on research and development; GERD/GDP, GERD as percentage of GDP; NA, not available.

**†**ISI Essential Science Indicators database (accessed 18 May 2004).

**‡**World Psychiatric Association Task Force (13).

§World Factbook (10).

**¶**UNESCO Science Report (11).

**††**Global Forum for Health Research (14).

**‡‡**Atlas Mental Health Project (12).

Table 1 also shows that the highest health expenditures as a proportion of the GDP are found for Argentina (9.5%), Croatia (9.0%) and South Africa (8.6%). The highest proportions of psychiatrists per 100 000 populations are shown for Argentina (13.6), Russia (13.3), the Czech Republic (12.1), and Hungary (9.0), and the lowest for India (0.2). The application of funding for research is shown as most substantial for the Czech Republic, Hungary, Poland and South Korea. The highest GDP per capita is shown for Kuwait, Singapore and United Arab Emirates. The highest number of publication per million inhabitants was found for Estonia (82.63) and Slovakia (80.66).

Of the 222 indexed psychiatric journals found in the 2007 survey of Medline and ISI/Thomson databases, only nine (4%) were published in middle-income countries, as shown in Table 1. No psychiatric journal from any low-income country was identified in Medline or ISI databases. The LAMIC journals presented in at least one of the two main databases are described in Table 2. Of the five journals from Central and Eastern Europe, two are indexed in both ISI/Medline databases (one from Croatia and one from Russia), and three others indexed in Medline (Hungary, Poland and Turkey). The four indexed journals published in Latin America include two from Brazil, indexed in both databases, one from Argentina indexed in the Medline, and the Mexican Salud Mental, indexed in ISI. Except for Latin America where three of the most productive countries (Argentina, Brazil and Mexico) present at least one journal indexed in the main databases, for Asia and Africa there was no clear relationship between indexation and the selected parameters displayed in Table 1, particularly the number of publications.

**2:** The psychiatric indexed journals from low- and middle-income countries in the two main databases

Title	Country	World Psychiatric Association Zone	Medline	Web of science
Vertex	Argentina	Southern South America	X	
Arquivos de Neuropsiquiatria	Brazil	Southern South America	X	X
Revista Brasileira de Psiquiatria	Brazil	Southern South America	X	X
Psychiatria Danubina	Croatia	Central Europe	X	
Psychiatria Hungarica	Hungary	Central Europe	X	
Salud Mental	Mexico	Mexico, Central America		X
Psychiatria Polska	Poland	Central Europe	X	
Zhurnal Nevrologii i Psikhiatrii	Russia	Eastern Europe	X	X
Turkish Journal of Psychiatry	Turkey	Southern Europe	X	

The WPA task force received 26 questionnaires from editors of LAMIC non-indexed journals (11 from Latin America, seven from Central Europe, four from Asia and four from Africa). The journals were then assessed by members of the committee applying similar criteria as those adopted by the Medline/Pubmed and ISI/Thomson databases. It was concluded by consensus of the Task Force that at least five might be ready for submissions in the two databases: i) three from the Asian region (China, India and South Korea); ii) one from Africa (South Africa); and iii) one from Brazil.

The WPA Task Force qualitative appraisal of the non-indexed LAMIC mental health journals revealed the following main reasons impeding journals to achieve indexation: i) insufficient number and/or quality of articles to meet periodicity (more than four issues per year); ii) a focus on local language; iii) lack of international representation in the editorial board and iv) focusing on quantity rather than quality, i.e. by working with low levels of rejection rates.

## Discussion

The search for indexed psychiatric journals in the two international databases found that nine journals out of 222 were from LAMIC, none of which were from a low-income country, or from Asia or Africa. However, the WPA Task Force which reviewed 26 non-indexed LAMIC journals concluded that five journals would likely meet criteria for indexation by Medline and/or ISI/Thomson database (three from the Asian region, one from Africa and one from Latin America). For Asia and Africa, there is no clear relationship between indexation and scientific productivity or selected social and economic characteristics in the countries. Taking into consideration the research capacity of these countries, if the five journals identified by the WPA task force were incorporated in the two international databases there would be more coherence in the distribution of indexed journals across the regions and across these countries.

There are several limitations to address in the findings of this paper. The criteria used by the ISI/Thomson are not clearly defined, and there is a small chance of misclassification of subjects, and/or countries of origin. It is also possible that mental health articles published in non-psychiatric journals, such as journals of public health and general medicine, were missed in the counting. The criterion of 100 counted publications by country over a 10-year period, for establishing scientific productivity was arbitrary, and may be regarded as a low threshold particularly for populous countries like India and China. It is likely that the list of non-indexed journals is incomplete; there may be many other LAMIC journals to be added to the list. However, it is demonstrated that despite a significant level of scientific activity shown by China, India, South Africa and South Korea, none of these countries, and indeed, no LAMIC in the African and Asian regions, is so far represented by a psychiatric journal in the main international databases. This is a further action to be added to the recommendations identified in a recent editorial (15), which pointed out some important steps to decrease the 10/90 divide in global mental health.
